# Primary care providers’ preferences for pay-for-performance programs: a discrete choice experiment study in Shandong China

**DOI:** 10.1186/s12960-024-00903-2

**Published:** 2024-03-12

**Authors:** Wencai Zhang, Yanping Li, BeiBei Yuan, Dawei Zhu

**Affiliations:** 1 Dong Fureng Institute of Economic and Social Development, Wuhan University, Luojia Hill, Wuhan, 430072 China; 2 Economics and Management School, Wuhan University, Luojia Hill, Wuhan, 430072 China; 3 China Center for Health Development Studies, Peking University, No. 38 Yueyuan Road, Haidian District, Beijing, 100191 China; 4 Department of Pharmacy Administration and Clinical Pharmacy, School of Pharmaceutical Sciences, Peking University, No. 38 Yueyuan Road, Haidian District, Beijing, 100191 China; 5 International Research Center for Medicinal Administration (IRCMA), Peking University, No. 38 Yueyuan Road, Haidian District, Beijing, 100191 China

**Keywords:** Pay for performance, Primary healthcare, Preferences, Discrete choice experiment, China

## Abstract

**Background:**

Pay-for-performance (P4P) schemes are commonly used to incentivize primary healthcare (PHC) providers to improve the quality of care they deliver. However, the effectiveness of P4P schemes can vary depending on their design. In this study, we aimed to investigate the preferences of PHC providers for participating in P4P programs in a city in Shandong province, China.

**Method:**

We conducted a discrete choice experiment (DCE) with 882 PHC providers, using six attributes: type of incentive, whom to incentivize, frequency of incentive, size of incentive, the domain of performance measurement, and release of performance results. Mixed logit models and latent class models were used for the statistical analyses.

**Results:**

Our results showed that PHC providers had a strong negative preference for fines compared to bonuses (− 1.91; 95%CI − 2.13 to − 1.69) and for annual incentive payments compared to monthly (− 1.37; 95%CI − 1.59 to − 1.14). Providers also showed negative preferences for incentive size of 60% of monthly income, group incentives, and non-release of performance results. On the other hand, an incentive size of 20% of monthly income and including quality of care in performance measures were preferred. We identified four distinct classes of providers with different preferences for P4P schemes. Class 2 and Class 3 valued most of the attributes differently, while Class 1 and Class 4 had a relatively small influence from most attributes.

**Conclusion:**

P4P schemes that offer bonuses rather than fines, monthly rather than annual payments, incentive size of 20% of monthly income, paid to individuals, including quality of care in performance measures, and release of performance results are likely to be more effective in improving PHC performance. Our findings also highlight the importance of considering preference heterogeneity when designing P4P schemes.

**Supplementary Information:**

The online version contains supplementary material available at 10.1186/s12960-024-00903-2.

## Introduction

Primary healthcare (PHC) has been identified as an integral part of achieving both the Sustainable Development Goals by 2030 as well as universal health coverage [1]. PHC provides integrated, accessible, and people-centered care that meets the health needs of individuals, families, and communities. However, the performance of PHC varies widely across different countries and regions [2]. However, PHC's performance varies widely across countries and regions due to various challenges, such as inadequate funding, poor infrastructure, shortages of healthcare workers, and limited access to essential medicines and technologies [3].

China’s Primary healthcare system is not an exception. PHC plays an increased role in providing care within the Chinese healthcare system due to its large population size, diverse demographics, geographic dispersion, limited resources, high disease burden, aging infrastructure, and lack of accessibility to specialty services for many individuals living there [4]. China has undergone a dramatic socio-economic and demographic transition. This transition has engendered a spectrum of challenges, including inadequate education and training for healthcare personnel, an aging workforce, suboptimal financial incentives, and poor disease prevention strategies [5].

Efforts are being made to improve the performance of PHC globally. Pay-for-Performance (P4P) scheme is one of strategies that are increasingly used to improve the performance of PCP [6]. P4P refers to a system where healthcare providers are paid based on the quality and efficiency of care they provide. Under this model, providers may receive bonuses or other incentives if their patients have good outcomes relative to an established benchmark. This is intended to encourage better performance from healthcare staff while also holding them accountable for patient satisfaction and healthcare costs. Due to the improved accuracy of performance measurement over the last 20 years [7], offering monetary rewards for better results appears reasonable.

Although there is agreement that P4P has the potential to enhance performance, the effectiveness of P4P has not been clearly established. Studies have yielded mixed or inconclusive results [8]. The key reason for the heterogeneity is the difference in designs, implementations, and settings [7, 9–11]. Previous literature reviews found many studies do not report the justification for the P4P program or important aspects related to P4P designs. They also identified several key characteristics in the design of P4P schemes, such as including healthcare professionals and other stakeholders in schemes' design, whom to incentivize, incentive type, size, and frequency.

The active involvement and support of healthcare providers is imperative for the successful implementation of P4P programs, as they can make significant contributions toward achieving P4P's objectives [12]. In addition, providers' concerns can offer insightful information to purchasers, enabling them to redesign programs in a manner that optimizes the positive impact on healthcare quality. Providers who do not value P4P programs may not be as driven to improve care [7]. In addition, unfair or unsatisfactory P4P program conditions could lead to reduced provider participation and program effectiveness [13]. The attitudes and perspectives of healthcare providers are therefore a determinant of P4P program effectiveness.

As healthcare providers are not only key stakeholders but also significant participants of P4P schemes, it is critical to better understand providers’ preferences for pay-for-performance schemes. Discrete choice experiments (DCEs) are a commonly used method to collect data on individual preferences [14]. By asking responders to choose between hypothetical goods or services with different attributes, DCEs provide a simplified version of real-life decision-making. Recently, there has been an increase in the use of DCEs to elicit the job preferences of health personnel [15].

The goal of this paper is to investigate the preferences of primary care providers for participating in P4P programs using DCEs. The study also aimed to investigate the extent of inter-individual preference heterogeneity present among the primary care providers.

## Methods

### Discrete choice experiments

In this study, DCEs were conducted to examine and quantify the preferences of primary care providers for P4P programs. The selection of attributes and their respective levels were based on a thorough review of existing literature and input from a group of experts. Six characteristics were identified for this purpose, with each character having up to four distinct levels. The attributes and their corresponding levels have been clearly delineated in Table 1. Below are the reasons for choosing these attributes and their levels:

1. Type of incentive: This attribute refers to the forms of financial incentive used in P4P schemes. There are two levels for this attribute—bonuses and fine. Fines tend to motivate behavioral change more than bonuses, but can undermine intrinsic motivation [10].
2. Whom to incentivize: Incentives can be provided to individuals (e.g., a doctor or nurse) or to groups (e.g., a group of healthcare workers in a PHC). Group-based P4P schemes are more likely to catalyze changes in group culture and management structures, while paying directly to individuals has the advantage of avoiding the problem of “free riders” [7].
3. Frequency of incentive: This attribute refers to how frequently they are evaluated and how frequently they are given. People tend to discount future gains at an increasing rate as time passes [16]. In P4P schemes, high frequency is more important if providers do not know how much improvement they are going to achieve.
4. Size of incentive: The size of incentive is represented by the proportion of income. According to Hahn, incentives might have different effects based on their size relative to salaries [17]. The proportion is respectively 10%, 20%, 40%, and 60% which is supposed to be consistent with local practice.
5. Domain of performance measurement: This attribute describes how “good performance” is measured. Healthcare visits refer to service volumes while quality of care refers the process quality of care, such as patient satisfaction, continuity of care and length of stay [11]. Benchmarks were set at the individual level.
6. Release of performance results: This attribute refers whether they know others’ performance and the amount of the reward. It may be possible to improve provider performance by releasing performance data [18].

**Table 1:** Attributes and levels

Attributes	Levels
Type of incentive	Bonuses^a^; Fines
Whom to incentivize	Individuals^a^; Groups
Frequency of incentive	Monthly^a^; quarterly; semiyearly; annually
Size of incentive (proportion of income)	10%^a^; 20%; 40%; 60%
Domain of performance measurement	Healthcare visits^a^; Healthcare visits & Quality of care
Release of performance results	Yes^a^; No

^a^reference level

DCEs' assumption P4P programs can be described by the combination of these attributes and attribute levels. The selection of six attributes with corresponding levels resulted in 256 potential service alternatives. To enhance the efficiency and decrease the burden on respondents during the survey process, we utilized an orthogonal experimental design through a mix-and-match algorithm to develop the choice sets with two blocks of eight tasks each [19]. This approach involves placing a collection of 16 alternatives derived from the orthogonal main-effect array taken from Kuhfeld’s (2009) into an urn. Additional sets of 16 alternatives are generated using the rotation method and placed into separate urns. A choice set is formed by randomly selecting one option from each urn, and this process is repeated until all 16 choice sets have been assigned without duplication. These 16 choice sets are randomly divided into 2 blocks. Participants were requested to select their preferred type of P4P scheme from the presented tasks. Each choice task described 2 alternative P4P scheme, and an opt-out choice (none of the above) was also included. By including ‘opt-out’, we can estimate participation rate and avoid overestimation of welfare values [20].

### Non-DCE variables

The survey also contained several questions on sociodemographic characteristics and work experience, including age, sex, education level, job title, permanent staff, any management position, years of practice, weekly working hours, monthly income, the proportion of fixed income, and work motivation. There is debate surrounding the “crowding out” effect of P4P schemes, and previous research shows that aligning incentives with providers’ motivation increases the odds of success [7]. The work motivation was measured using a well-validated psychometric scale based on self-determination theory (SDT) [21]. In the mid-1980s, SDT was introduced as a general framework for human motivation and has been extensively studied and refined ever since [22]. According to SDT, motivation has five main components: external regulation, which is based on a practical goal of behavior; introjected regulation, which is based on self-esteem, prestige, or obligation; identified regulation, which is based on the importance of the job; integrated regulation, which is based on the congruence between personal and professional goals and values; and intrinsic motivation, which comes from within the person. The scale we used merged integrated and identified regulation and split external regulation into social and economic subfactors for optimal model fit [21]. The scale consists of a 15-item inventory that evaluates five dimensions of work motivation: intrinsic motivation (IM, 3 items), integrated/identified regulation (IDEN, 3 item), introjected regulation (INTRO, 2 items), external regulation-social (EXT-S, 3 item), and external regulation-economic (EXT-E, 4 items). More details can be found in Lohmann et al. [21]. Each item is rated on an 11-point Likert scale, with 0 indicating complete disagreement and 10 indicating complete agreement. The scores for each dimension were determined by summing the individual item scores and then standardizing them to the sample mean.

### Sampling and participants

This study is a component of a larger cross-sectional survey on the integrated primary health service system in Weifang city, Shandong province. We used a stratified random sampling method to recruit primary care providers in six counties in Weifang City. Data were collected by face-to-face interview using questionnaires, and participants were eligible if they met the following criteria: (1) currently working in primary care, and (2) had experience with P4P programs. According to Johnson and Orme [23], a sample size of 125 participants was deemed sufficient to achieve statistical power. However, since our sample consists of four subgroups, the minimum sample size should be 500. A total of 882 primary healthcare (PHC) providers participated in the study which meet the minimum sample size requirements.

### Statistical analyses

Descriptive statistics were employed to examine the characteristics of the respondents. Mixed logit models were utilized to derive mean utilities (preferences) and standard deviations of random coefficients for the population. The utility of an individual $n$ choosing alternative $i$ (with $k$ attributes) at the $t$ choice set is expressed as follows:

$$U_{{{\text{nit}}}} = V_{{{\text{nit}}}} + \varepsilon_{{{\text{nit}}}} = \mathop \sum \limits_{k = 1}^{k} \beta_{k} x^{\prime}_{{{\text{nit}}k}} + \varepsilon_{{{\text{nit}}}} ,$$

where ${V}_{{\text{nit}}}$ is the deterministic part of the utility, and $\beta$ is a vector of estimated coefficients which signify the relative weight of preference for a particular attribute comparison, and represents the relative contribution of the attribute level to the utility that respondents assign to an alternative. Positive coefficients denote favorable preferences, while negative coefficients denote unfavorable preferences.

We also fit latent class models (LCM) to identify different segments of individuals with distinct preferences. The LCM assumes that individuals belong to one of $q$ classes, each with a different set of coefficients ${\beta }_{q}$. The probability of choosing alternative $i$ for individual $n$ is then:

$$\Pr \left( {{\text{choice}} = 1} \right) = \mathop \sum \limits_{q} {\text{Pr}}({\text{choice}} = i|\beta_{q} )\pi_{q} ,$$

where ${\pi }_{q}$ is the probability of being in class $q$*.* The choice probability within a class $q$ is estimated using conditional logit:

$$\Pr \left( {{\text{choice}}_{n} = i|\beta_{q} } \right) = \frac{{e^{{v\left( {\beta_{q} ,x_{i} } \right)}} }}{{\mathop \sum \nolimits_{j} e^{{v\left( {\beta_{q} ,x_{j} } \right)}} }}.$$

We select the optimal number of classes based on the Akaike and Bayesian information criteria (AIC and BIC). The LCM was determined to have four classes based on the significantly better AIC and BIC values compared to the two-class, three-class, and five-class models.

## Results

### Characteristics of participants

A total of 882 primary healthcare (PHC) providers participated in the study, with a mean age of 38.8 years (SD = 9.1). Among them, 68.5% were female and 31.5% were male. The majority of the participants were married (83.8%) and had a middle school education or below (46.5%). About two-thirds of the participants held junior job titles (66.4%) and were not in any management positions (90.5%). The mean years of practice and years of service in the institution were 16.4 (SD = 9.9) and 14.1 (SD = 9.9), respectively. The majority of the participants worked less than or equal to 50 h per week (60.2%) and had a monthly income of less than or equal to 5000 RMB (70.5%). Most participants (57.4%) had a proportion of fixed income less than or equal to 80%. In terms of work motivation, the mean scores for intrinsic motivation, integrated/identified regulation, introjected regulation, external regulation-social, and external regulation-economic were 19.8 (SD = 9.2), 22.2 (SD = 8.5), 14.4 (SD = 5.8), 20.8 (SD = 8.7), and 29.1 (SD = 10.5), respectively (Table 2).

**Table 2:** Participant characteristics

Characteristics	*N* (%)
Age^a^	38.8 (9.1)
Sex	
Female	604 (68.5)
Male	278 (31.5)
Married	
No	143 (16.2)
Yes	739 (83.8)
Education level	
Middle school or below	410 (46.5)
Bachelor’s degree or higher	472 (53.5)
Job title	
Juniors	586 (66.4)
Senior or intermediate	296 (33.6)
Permanent staff	
No	530 (60.1)
Yes	352 (39.9)
Any management position	
No	798 (90.5)
Yes	84 (9.5)
Years practiced	
≤ 15	409 (46.4)
> 15	473 (53.6)
Years serviced in this institution	
≤ 15	512 (58.1)
> 15	370 (42.0)
Weekly working hours	
≤ 50	531 (60.2)
> 50	351 (39.8)
Monthly income	
≤ 5000	622 (70.5)
> 5000	260 (29.5)
The proportion of fixed income (%)	
≤ 80	506 (57.4)
> 80	376 (42.6)
Work motivation (original score)^a^	
Intrinsic motivation (IM, range for 0 to 30)	19.8 (9.2)
Integrated/identified regulation (IDEN, range for 0 to 30)	22.2 (8.5)
Introjected regulation (INTRO, range for 0 to 20)	14.4 (5.8)
External regulation-social (EXT-S, range for 0 to 30)	20.8 (8.7)
External regulation-economic (EXT-E, range for 0 to 40)	29.1 (10.5)

^a^Mean and SD are reported for continuous variable

### Mean preferences

**Fig. 1:**
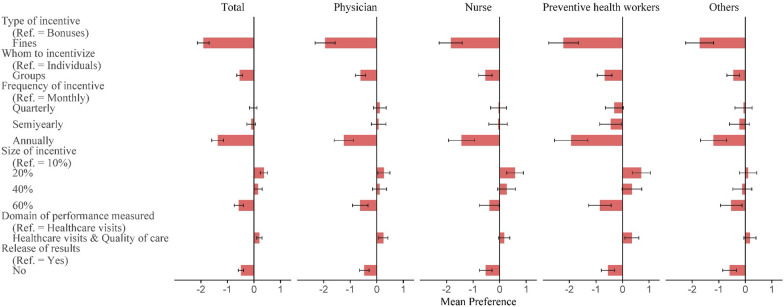
Mean preferences (relative utilities) for P4P programs, in total population and subgroups

The opt-out option was chosen in 17.02% of the choice sets. The study results for mean preference weights (estimated coefficient represents the relative contribution of the attribute level to the utility) for P4P programs among the total population and subgroups are shown in Fig. 1 (Additional file 1: Table S1). Negative preference for fines compared to bonuses (− 1.91; 95%CI − 2.13 to − 1.69) was the strongest preference across attributes, followed by a negative preference for annual incentive payments compared to monthly (− 1.37; 95%CI − 1.59 to − 1.14). PHC providers also exhibited a negative preference for incentive size of 60% of monthly income (− 0.57; 95%CI − 0.74 to − 0.40), group incentives (− 0.54; 95%CI − 0.665 to − 0.43), and non-release of performance results (− 0.49; 95%CI − 0.60 to − 0.39). An incentive size of 20% of monthly income was preferred over 10% (0.38; 95%CI 0.24 to 0.51), and responders favored the inclusion of quality of care in performance measures rather than healthcare visit measures only (0.20; 95%CI 0.10 to 0.30). The mixed logit model showed significant variation in preferences, indicated by standard deviations for multiple attributes.

Subgroup analysis revealed that preferences were largely consistent across various professions, although nurses and other healthcare professionals exhibited less susceptibility to the inclusion of quality-of-care in performance measures compared to measures solely based on healthcare visits.

### Latent class analysis

**Fig. 2:**
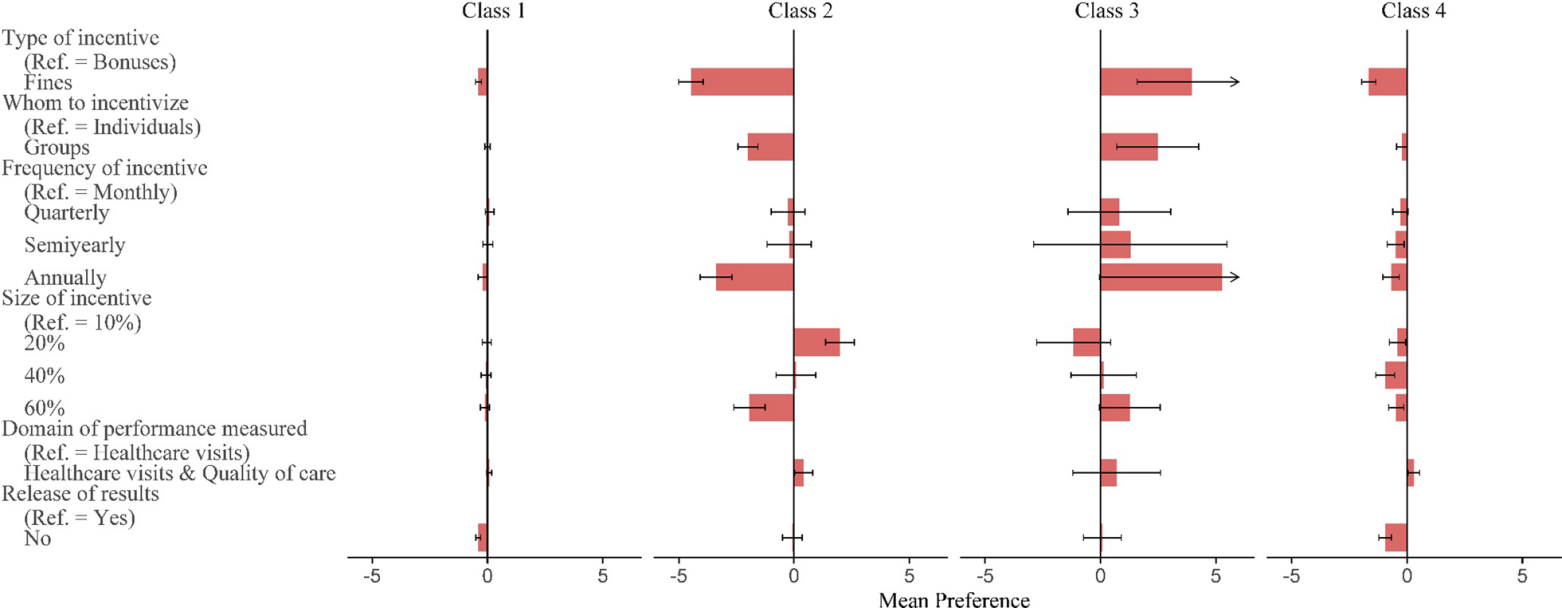
Preferences (relative utilities) for P4P programs, by latent class membership group

The latent class analysis revealed that PHC providers could be classified into four distinct classes with different preferences for P4P schemes (Fig. 2). Class 1 (21.8% membership probability) had minimal influence from most attributes, exhibiting small negative preferences for fines, annual incentive payments, and non-release of performance results. Class 2 (46.2% membership probability) had strong negative preferences for fines, annual incentive payments, group incentives, and incentives equivalent to 60% of monthly income. Class 3 (10.4% membership probability) showed nearly opposite preferences to Class 2 for these attributes. Finally, Class 4 (21.7% membership probability) exhibited similar preferences to Class 2, but with smaller weights.

The study found that various demographic and job-related factors had the greatest impact on determining class membership, including age, marital status, education level, job title, permanent staff status, years practiced, weekly working hours, monthly income, the proportion of fixed income, IM, IDEN, and EXT-E (Table 3). Compared to membership in Class 2, providers with a bachelor’s or higher degree, junior job title, years practiced > 15, monthly income > 5000 CNY, and lower in EXT-E were more likely to be in Class 1. Respondents who reported years practiced > 15 and a proportion of fixed income > 80% were less likely to be in Class 3. Providers who were younger in age, married, worked > 50 weekly hours, had monthly income > 5000, had higher IDEN, and had lower IM were more likely to be in Class 4. Predicted number and percentage of healthcare providers by professionals and class are presented in Additional file 1: Table S2, and there is no significant difference between professionals.

**Table 3:** Predictors of latent class membership for Class 1 (21.8%), 3 (10.4%), and 4 (21.7%) to Class 2 (46.2%), RRR (95%CI)

	Class 1 VS Class 2	Class 3 VS Class 2	Class 4 VS Class 2
Age	0.97 (0.93, 1.01)	0.99 (0.95, 1.04)	0.96 (0.93, 1.00)*
Male	1.08 (0.71, 1.65)	0.92 (0.54, 1.56)	0.76 (0.51, 1.15)
Married	0.88 (0.48, 1.65)	0.92 (0.43, 1.96)	2.07 (1.06, 4.04)*
Bachelor’s degree or higher	2.11 (1.30, 3.44)**	0.58 (0.32, 1.08)	1.28 (0.80, 2.06)
Senior or intermediate	0.52 (0.28, 0.97)*	1.16 (0.55, 2.45)	0.63 (0.36, 1.12)
Permanent staff	0.79 (0.45, 1.40)	1.76 (0.88, 3.52)	1.49 (0.88, 2.52)
Any management position	1.23 (0.62, 2.43)	1.36 (0.57, 3.23)	1.28 (0.68, 2.40)
Years practiced > 15	2.53 (1.22, 5.24)*	0.34 (0.12, 0.96)*	1.25 (0.61, 2.56)
Years serviced in this institution > 15	0.58 (0.33, 1.05)	1.92 (0.74, 5.01)	1.16 (0.64, 2.10)
Weekly working hours > 50	1.00 (0.67, 1.49)	1.15 (0.70, 1.90)	1.49 (1.03, 2.18)*
Monthly income > 5000	2.15 (1.16, 3.98)*	1.12 (0.52, 2.41)	1.82 (1.04, 3.19)*
The proportion of fixed income > 80%	1.04 (0.70, 1.54)	0.56 (0.33, 0.93)*	0.90 (0.62, 1.31)
IM	1.21 (0.80, 1.81)	1.12 (0.67, 1.89)	0.65 (0.46, 0.91)*
IDEN	1.12 (0.67, 1.88)	0.73 (0.37, 1.41)	1.96 (1.27,3.03)**
INTRO	0.81 (0.47, 1.41)	1.25 (0.61, 2.56)	1.03 (0.64, 1.65)
EXT-S	1.32 (0.78, 2.22)	0.93 (0.50, 1.76)	0.71 (0.46, 1.09)
EXT-E	0.66 (0.50, 0.86)**	0.89 (0.64, 1.23)	1.03 (0.79, 1.33)
Constant	0.84 (0.26, 2.68)	0.48 (0.13, 1.83)	0.65 (0.21, 2.05)

Class 2 was treated as the base outcome; *p < 0.05, **p < 0.01, ***p < 0.001

*IM* intrinsic motivation, *IDEN* integrated/identified regulation, *INTRO* introjected regulation, *EXT-S* external regulation-social, *EXT-E* external regulation-economic

## Discussion

This DCE study analyzed the preferences of primary healthcare providers for P4P programs in China. The results showed that providers had a strong negative preference for fines compared to bonuses and an annual payment of incentives compared to monthly. Providers also showed negative preferences for incentive size of 60% of monthly income, group incentives, and non-release of performance results. On the other hand, an incentive size of 20% of monthly income and including quality of care in performance measures were preferred. The study identified four distinct classes of providers with different preferences for P4P schemes. Class 2 and Class 3 valued most of the attributes differently, while Class 1 and Class 4 had a relatively small influence from most attributes. The results suggest that P4P programs need to consider the diversity of provider preferences and the potential impact of provider characteristics on their acceptance and effectiveness.

The study results demonstrated that PHC providers had a stronger negative preference for financial penalties than rewards, which is consistent with previous research showing that loss-based incentive schemes are often perceived as unfair and unacceptable [24]. While financial withholding might enhance performance more effectively than positive rewards based on loss aversion theory, it could lead to opportunistic behavior and increased incentives for undesirable conduct [25]. Performance outcomes are dependent on accurate measuring and reporting. These issues are acknowledged in relation to financial penalties, and it also suites in rewards. Performance can be measured by outcomes that are assumed to have occurred and are generally undesirable, such as adverse events or quality failures. However, these outcomes may not reflect the actual occurrence or the quality of care, as they may depend on factors such as reporting practices, definitions, or patient characteristics. To develop an effective incentive program, it is crucial to strike a balance between rewards and penalties, while considering the behavioral and motivational factors that drive healthcare providers.

One of the main findings of this paper is that the frequency of payments was the second most important attribute in the choice experiment and that respondents exhibited a negative preference for a longer time lag between payments. This result can be interpreted in terms of time preference and risk preference. Time preference refers to the degree to which people prefer to receive payments sooner rather than later, and risk preference refers to the degree to which people are willing to accept uncertainty in the amount or timing of payments [26]. A longer time lag implies a higher discount rate and a higher risk premium, which reduce the expected utility of the payments. Therefore, respondents who have a higher time preference or a higher risk aversion would prefer more frequent and immediate payments. More importantly, the shorter time lags and the regular feedback might have motivated the physicians more than a one-time lump sum incentive. A randomized controlled trial suggests that quarterly payments with quarterly reports were more effective than annual payments with yearly reports [27].

The results of this study suggest that providers give greater weight to incentive size of 20% of monthly income, individual incentives, and release of performance results when deciding whether to participate in the P4P scheme. Previous research revealed that the relative importance of these factors may vary depending on the context and design of the P4P scheme [11]. For example, in settings where providers have low baseline income or face high financial risk, incentive size may be more influential than in settings where providers have higher income or lower risk [28, 29]. In the context of our study setting, an incentive size of 20% of monthly income may be a suitable incentive size. Similarly, individual incentives may be more effective than group incentives in settings where providers have more autonomy and accountability, while group incentives may foster teamwork and coordination in settings where providers work interdependently [30]. Finally, the release of performance results may have different effects on provider reputation and competition depending on the level of transparency and public awareness of the P4P scheme.

Although most professions had similar preferences, the LCM identified four distinct classes of providers with different preferences for P4P schemes. Class 2 was the dominant class, accounting for 46.2% of the providers. Compared to the dominant class, membership in Class 1 was driven by higher education level, junior job title, longer practiced year, higher monthly income, and lower external regulation-economic factors and needed other non-financial incentives. Similarly, financial incentives may have a small incentive to membership in Class 4. Last and most importantly, membership in Class 3 showed almost opposite preferences to the dominant class, even though they only accounted for 10 percent of providers. There is no significant difference in predicted Class between professionals. It is crucial to find effective incentives to offset their utility losses through P4P schemes.

Our study has several limitations. First, we selected the most relevant attributes based on the literature and interviews with experts in the field of P4P schemes, which is unable to include all attributes of P4P schemes. Second, our sample only included PHC providers from one city in China. Therefore, further studies are needed to confirm the generalization of our findings, especially when testing for different geographical locations. Third, preferences may change over time as providers learn more about the P4P programs and their consequences. Therefore, the results of this study may not reflect the actual behavior of providers when faced with different P4P schemes in practice.

Despite these limitations, we believe that this is the first study of PHC providers’ preference for P4P schemes, which provides new information on PHC providers’ preferences for participating in P4P programs. Based on these findings, a policy suggestion is to design P4P schemes that offer individual bonuses based on quality and quantity indicators, pay incentives monthly or more frequently, and make performance results publicly available. Moreover, policymakers should consider the diversity of provider preferences and characteristics when designing and implementing P4P schemes, and tailor the schemes to different segments of providers based on their motivation, experience, and location.

## Conclusion

In conclusion, this study provides important insights into the preferences of primary care providers for P4P programs. The findings suggest that the type of incentive was the most important attribute in PHC providers’ preferences for P4P schemes, followed by incentive frequency, size, whom to incentivize, the release of performance results, and the domain of performance measurement. In addition, we found evidence of preference heterogeneity. To improve the quality and efficiency of primary healthcare in China, policymakers should design and implement P4P schemes that account for the preferences and characteristics of healthcare providers.

### Supplementary Information

**Additional file 1: Table S1.** Preferences (relative utilities) for P4P programs, in total population and subgroups. **Table S2.** Predicted number and percentage of healthcare providers by professionals and class

## Data Availability

All data generated and analyzed during this study are included in this published article.
