# Supplementary material for: Primary care providers’ preferences for pay-for-performance programs: a discrete choice experiment study in Shandong China
Source: Hum Resour Health. 2024 Mar 12;22:20. doi: 10.1186/s12960-024-00903-2 (PMC10936064; doi:10.1186/s12960-024-00903-2)
Supplement: Supplementary file 1 — Additional file 1: Table S1. Preferences (relative utilities) for P4P programs, in total population and subgroups. Table S2. Predicted number and percentage of healthcare providers by professionals and class [file 12960_2024_903_MOESM1_ESM.docx]

Table S1 Preferences (relative utilities) for P4P programs, in total population and subgroups

|  | Total | | Physician | | Nurse | | Preventive health workers | | Others | |
| --- | --- | --- | --- | --- | --- | --- | --- | --- | --- | --- |
| Attribute | β means (SE) | S.D. (SE) | β means (SE) | S.D. (SE) | β means (SE) | S.D. (SE) | β means (SE) | S.D. (SE) | β means (SE) | S.D. (SE) |
| Constant | 7.029***  (0.439) | 6.146***  (0.373) | 7.253***  (0.842) | 5.791***  (0.536) | 7.884***  (1.174) | 6.571***  (1.199) | 8.017***  (1.030) | 6.686)***  (0.828 | 5.755***  (0.816) | 4.879***  (0.610) |
| Type of incentive (Ref. = Bonuses) | | |  |  |  |  |  |  |  |  |
| Fines | -1.910  (0.112) | 2.010  (0.110) | -1.946  (0.193) | 2.061  (0.210) | -1.843  (0.222) | 2.113  (0.291) | -2.228  (0.284) | 2.385  (0.294) | -1.721  (0.269) | 2.250  (0.283) |
| Whom to incentivize (Ref. = Individuals) | | |  |  |  |  |  |  |  |  |
| Groups | -0.540***  (0.055) | 0.710***  (0.085) | -0.615***  (0.097) | 0.765***  (0.154) | -0.536***  (0.130) | 0.797***  (0.182) | -0.674***  (0.144) | 0.999***  (0.214) | -0.455***  (0.122) | -0.629***  (0.202) |
| Frequency of incentive (Ref. = Monthly) | | |  |  |  |  |  |  |  |  |
| Quarterly | -0.024  (0.071) | -0.014  (0.088) | 0.116  (0.120) | -0.037  (0.160) | -0.043  (0.152) | -0.016  (0.191) | -0.305  (0.171) | -0.021  (0.179) | -0.067  (0.165) | -0.121  (0.269) |
| Semiyearly | -0.105  (0.084) | 0.016  (0.102) | 0.070  (0.140) | -0.089  (0.205) | -0.061  (0.179) | 0.030  (0.200) | -0.445*  (0.210) | 0.058  (0.230) | -0.219  (0.192) | 0.139  (0.306) |
| Annually | -1.367***  (0.113) | 1.696***  (0.131) | -1.244***  (0.184) | 1.603***  (0.225) | -1.437***  (0.250) | 1.605***  (0.298) | -1.932***  (0.321) | -2.278***  (0.329) | -1.198***  (0.252) | 1.908***  (0.339) |
| Size of incentive (Ref. = 10%) | |  |  |  |  |  |  |  |  |  |
| 20% | 0.375***  (0.070) | 0.383*  (0.192) | 0.270*  (0.117) | 0.374  (0.321) | 0.583***  (0.162) | -0.653*  (0.305) | 0.708***  (0.175) | 0.456  (0.351) | 0.106  (0.164) | -0.563  (0.341) |
| 40% | 0.156  (0.080) | -0.038  (0.127) | 0.104  (0.135) | 0.013  (0.234) | 0.268  (0.171) | 0.143  (0.274) | 0.360  (0.190) | -0.010  (0.289) | -0.114  (0.183) | -0.115  (0.339) |
| 60% | -0.571***  (0.089) | 0.953***  (0.116) | -0.624***  (0.149) | -0.859***  (0.235) | -0.390*  (0.191) | 1.072***  (0.264) | -0.850***  (0.217) | -0.872**  (0.266) | -0.531*  (0.208) | 1.148***  (0.247) |
| Domain of performance measured (Ref. = Healthcare visits) | | | | |  |  |  |  |  |  |
| Healthcare visits & Quality of care | 0.202***  (0.051) | 0.056  (0.088) | 0.245**  (0.088) | 0.002  (0.130) | 0.173  (0.109) | -0.034  (0.206) | 0.355**  (0.134) | -0.046  (0.310) | 0.178  (0.117) | 0.029  (0.175) |
| Release of results (Ref. = Yes) | |  |  |  |  |  |  |  |  |  |
| No | -0.492***  (0.053) | 0.072  (0.115) | -0.471***  (0.091) | 0.150  (0.192) | -0.525***  (0.122) | -0.418  (0.278) | -0.544***  (0.128) | -0.119  (0.392) | -0.592***  (0.132) | 0.186  (0.250) |

S.D. = standard deviation, ***Denotes P < 0.01, **P < 0.05, *P < 0.10

Table S2 Predicted number and percentage of healthcare providers by professionals and class

|  | Class 1 | Class 2 | Class 3 | Class 4 |
| --- | --- | --- | --- | --- |
| Physician | 66(20.50) | 154(47.83) | 30(9.32) | 72(22.36) |
| Nurse | 45(22.17) | 101(49.75) | 20(9.85) | 37(18.23) |
| Preventive health workers | 36(19.25) | 94(50.27) | 18(9.63) | 39(20.86) |
| Others | 39(22.94) | 64(37.65) | 23(13.53) | 44(25.88) |
| Total | 186(21.09) | 413(46.83) | 91(10.32) | 192(21.77) |
